# Frontal lobe microglia, neurodegenerative protein accumulation, and cognitive function in people with HIV

**DOI:** 10.1186/s40478-022-01375-y

**Published:** 2022-05-07

**Authors:** Jacinta Murray, Gregory Meloni, Etty P. Cortes, Ariadna KimSilva, Michelle Jacobs, Alyssa Ramkissoon, John F. Crary, Susan Morgello

**Affiliations:** 1 Department of Neurology, The Icahn School of Medicine at Mount Sinai, Box 1137, Mount Sinai Medical Center, New York City, NY 10029 USA; 2 Department of Neuroscience, The Friedman Brain Institute, The Icahn School of Medicine at Mount Sinai, New York City, NY USA; 3 Department of Artificial Intelligence and Human Health, Ronald M. Loeb Center for Alzheimer’s Disease, The Icahn School of Medicine at Mount Sinai, New York City, NY USA; 4 Department of Pathology, The Icahn School of Medicine at Mount Sinai, New York City, NY USA

**Keywords:** Microglia, HIV, Amyloid beta, Tau

## Abstract

Microglia are implicated in Alzheimer’s Disease (AD) pathogenesis. In a middle-aged cohort enriched for neuroinflammation, we asked whether microgliosis was related to neocortical amyloid beta (A$\upbeta$) deposition and neuronal phosphorylated tau (p-tau), and whether microgliosis predicted cognition. Frontal lobe tissue from 191 individuals autopsied with detectable (HIV-D) and undetectable (HIV-U) HIV infection, and 63 age-matched controls were examined. Immunohistochemistry (IHC) was used to evaluate A$\upbeta$ plaques and neuronal p-tau, and quantitate microgliosis with markers Iba1, CD163, and CD68 in large regions of cortex. Glia in the A$\upbeta$ plaque microenvironment were quantitated by immunofluorescence (IF). The relationship of microgliosis to cognition was evaluated. No relationship between A$\upbeta$ or p-tau accumulation and overall severity of microgliosis was discerned. Individuals with uncontrolled HIV had the greatest microgliosis, but fewer A$\upbeta$ plaques; they also had higher prevalence of APOE $\upvarepsilon$4 alleles, but died earlier than other groups. HIV group status was the only variable predicting microgliosis over large frontal regions. In contrast, in the A$\upbeta$ plaque microenvironment, APOE $\upvarepsilon$4 status and sex were dominant predictors of glial infiltrates, with smaller contributions of HIV status. Cognition correlated with large-scale microgliosis in HIV-D, but not HIV-U, individuals. In this autopsy cohort, over large regions of cortex, HIV status predicts microgliosis, whereas in the A$\upbeta$ plaque microenvironment, traditional risk factors of AD (APOE $\upvarepsilon$4 and sex) are stronger determinants. While microgliosis does not predict neurodegenerative protein deposition, it does predict cognition in HIV-D. Increased neuroinflammation does not initiate amyloid deposition in a younger group with enhanced genetic risk. However, once A$\upbeta$ deposits are established, APOE $\upvarepsilon$4 predicts increased plaque-associated inflammation.

**Supplementary Information:**

The online version contains supplementary material available at 10.1186/s40478-022-01375-y.

## Introduction

Converging lines of evidence support a central role for microglial cells in the pathogenesis of late onset Alzheimer’s Disease (AD). Many genetic susceptibility loci fall in pathways governing innate immunity and myeloid differentiation, and disease-associated antigenic, transcriptomic, and morphologic patterns of microglial cell activation have been observed in anatomic relation to amyloid beta (A$\upbeta$) plaques and in some cases, phosphorylated-tau (p-tau) pathologies in human brain [1–8]. However, there is significant debate as to the timing and nature of microglial cell involvement in disease evolution. It is unclear whether microglial cells initiate or actively contribute to the evolution of AD-associated neuropathology and clinical dysfunction, whether they participate in reactive, and potentially ameliorative processes to limit damage induced by neurodegeneration, or whether they are related to other clinical and pathologic factors extant at the time point of observation. While animal models can explicitly examine the time course of AD neuropathology and its relation to neuroinflammation, there are many impediments to similar studies in humans. While positron emission tomography (PET) techniques have been developed for the identification of A$\upbeta$ and p-tau through the adult lifespan, the target ligand used for identification of microglia, 18 kDa translocator protein (TSPO), is also expressed in astrocytes, endothelia and smooth muscle [9]. Furthermore, while generally showing AD-related increases in neuroinflammation, the TSPO-PET literature is variable with respect to the timing and location of these abnormalities [10]. Human post-mortem brain studies are similarly contradictory. Using aged as well as “high pathology” controls with AD histology and normal mentation, some authors have interpreted their analyses as supportive of late, reactive microglial phenomena; others, of neuroinflammation that contributes to pathogenesis by virtue of intrinsic microglial dysfunction and senescence, or by way of quantitative differences in AD pathology-associated microglial activation that correspond to clinically relevant deficits [2, 3, 6, 11].

Another less-explored issue with regard to AD neuropathology is whether co-morbid diseases modify the relationship between abnormal protein accumulation, microglial cell activation, and clinical deficit. Multiple groups have demonstrated a complex relationship between AD and non-AD brain pathologies and clinical impairments, with “person-specific” combinations of vascular and other neurodegenerative phenomena found at autopsy in demented individuals [12–14]. The relevance of this heterogeneity to neuroinflammation is unclear. Furthermore, co-morbidities potentially relevant to neuroinflammation need not be intrinsic to brain; some have observed that diseases characterized by persistent systemic immune activation increase the risk of cognitive impairments in the elderly, and that disease-modifying anti-inflammatory therapies may decrease this risk [15]. In keeping with the concept of extrinsic modifiers, one study has demonstrated that systemic infection at the time of death is associated with altered expression of microglial activation markers in the brains of individuals dying with late stage AD [16].

Neuropathologic studies of people with HIV (PWH) provide a unique opportunity to examine the relationship between microglial cell activation, clinical deficit, and the abnormal deposition of AD-related proteins A$\upbeta$ and p-tau in the setting of a disorder with known immunologic consequence. Systemic HIV infection results in a diverse and complex neuroinflammatory response, one component of which is microgliosis—the reaction of microglial cells to an abnormal stimulus, often through increases in number. In PWH, microgliosis has been associated with a cognitive disorder, and the qualitative and quantitative aspects of microglial cell activation in natural history disease are well-established. HIV-associated microglial responses have been documented in human brain with diverse methodologies, including immunohistochemical (IHC) studies to detect CD68 (an endosomal/lysosomal marker activated through Toll-like receptor 4 stimulation), CD163 (a scavenger receptor reflecting activation biased to an “M2” phenotype), and Iba1 (ionized calcium binding adaptor molecule 1, a pan-microglial marker) [17–19]

By controlling HIV replication, combination antiretroviral therapy (cART) can ameliorate, but not extirpate, systemic and brain inflammation, with increased lifespan into decades in which the earliest manifestations of AD neuropathology are thought to occur [20, 21]. Thus, middle-aged HIV cohorts can provide insight into how chronic immune stimulus impacts the initial manifestations of AD neuropathologies. Utilizing the autopsy cohort of the Manhattan HIV Brain Bank (MHBB), we previously demonstrated that the duration of HIV disease constitutes an independent risk for cortical A$\upbeta$ deposition, that in PWH replaces the most important time dependent variable known to predict AD: biologic age [22]. Herein, we examine this predominantly middle-aged cohort to determine if activated brain microglia are related to the abnormal deposition of A$\upbeta$ and neuronal p-tau in large regions of frontal cortex and in the amyloid plaque microenvironment, both in bivariate and multivariate analyses. We examine whether HIV-associated variables including cART treatment, other comorbid diseases, or more traditional AD risk factors are related to microglial cell populations in these large and small neocortical areas. We further explore the relevance of microglial cell populations to cognitive dysfunction in prospectively followed individuals.

## Materials and methods

### Patient population

HIV positive and HIV negative (HIV-neg) MHBB donors were autopsied between the years 1999 and 2019. The Icahn School of Medicine at Mount Sinai (ISMMS) Institutional Review Board provides oversight for the MHBB, and all donations follow approved forms of consent. This sample was representative of the larger MHBB population, details of which have been described previously [22]. Clinical information about co-morbid diseases, therapies, and for PWH, immunovirologic status, was obtained through patient participation in the prospective, observational MHBB study, and/or through medical record review at the time of autopsy. For all analyses, PWH whose terminal plasma HIV load was greater than 49 copies/ml (log 1.69) were classified as detectable (HIV-D), and those at or below this threshold, undetectable (HIV-U).

### Cognitive characterization

A total of 135 of the 191 PWH in this analysis underwent cognitive assessments in the MHBB study. A comprehensive neuropsychologic test battery was administered, as previously described [23]. In brief, up to 14 tests are administered and scored with demographically and educationally-adjusted norms to calculate a global T score and T scores for the following putative cognitive domains: motor function, speed of information processing, attention/working memory, learning (memory encoding), recall (memory retrieval), verbal fluency, and abstraction/executive function. T scores are normally distributed with a mean of 50 and standard deviation (SD) of 10; impairment is diagnosed with scores < 40. The last test battery administered was used for analysis, occurring at a median of 162 days [95, 267] interquartile range (IQR) prior to demise.

### Brain processing and staining

**Fig. 1:**
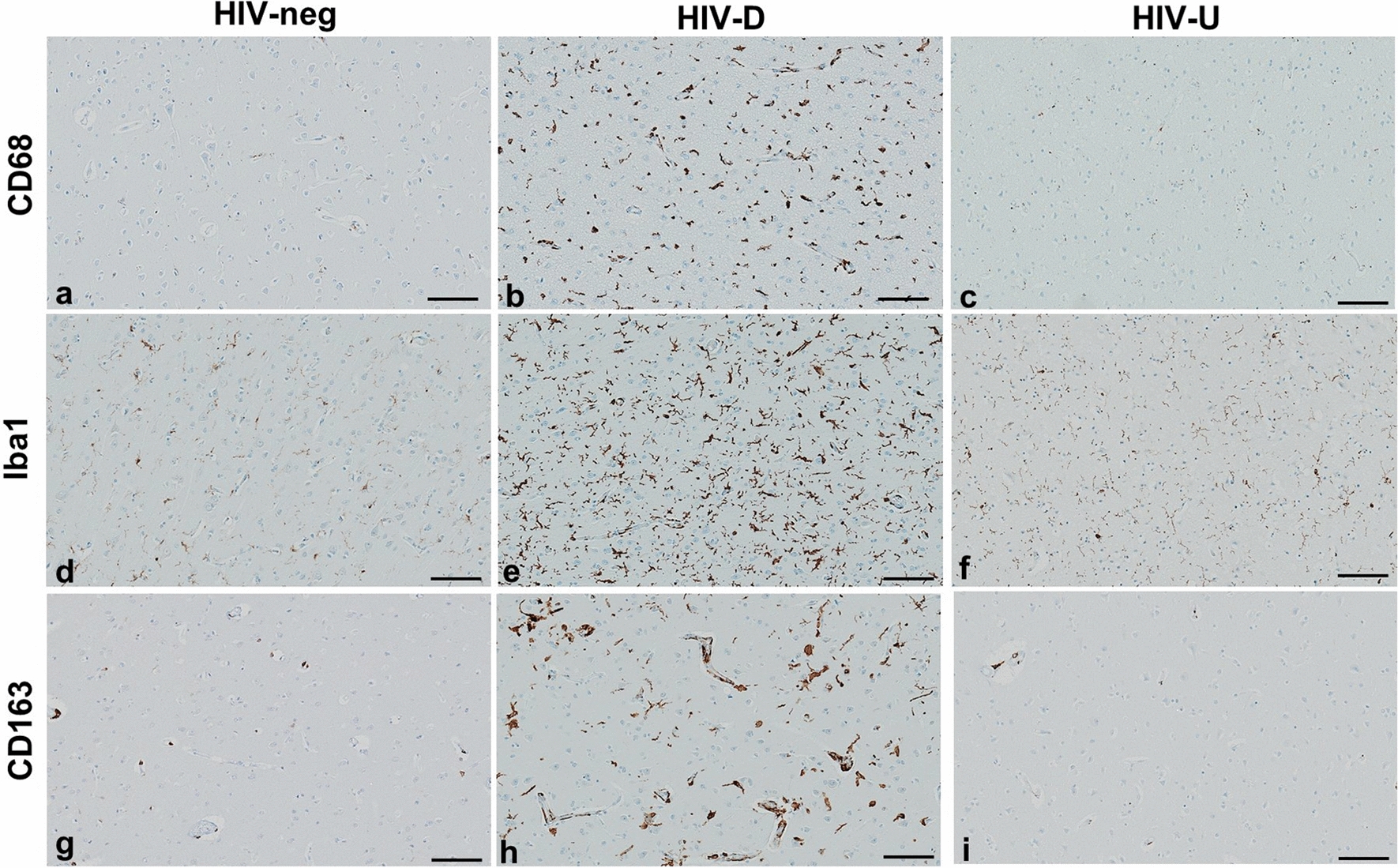
Representative immunohistochemistry for frontal cortex microglial markers in the 3 patient groups (HIV-negative, HIV-detectable viral load, HIV-undetectable viral load). Stains for CD68 **a**-**c**, Iba1 **d**-**f**, and CD163 **g**-**i**, demonstrate the increased microglial presence in HIV-D compared to the other groups. Diaminobenzidene chromogen, hematoxylin counterstain. Scale bar 100um

At autopsy, half the brain is frozen and half formalin-fixed for routine processing and sectioning. A minimum of 50 tissue blocks for histologic assessment by a board-certified neuropathologist (SM) are obtained as described [24]. For this analysis, blocks of mid frontal gyrus (predominantly Brodman area 9) were sectioned at 5 microns (um) and stained with hematoxylin and eosin, Luxol fast blue, and Bielschowsky techniques. Immunohistochemistry (IHC) was performed on a Ventana Benchmark XT autostainer (Roche Tissue Diagnostics, Tucson, AZ), with primary antibodies to detect A$\upbeta$ (clone 4G8, 1:8000 dilution, catalog number (cat#) 800701, Biolegend, San Diego, CA), p-tau (clone AT8, 1:1000 dilution, cat#MN1020, Thermo Fisher Scientific, Waltham, MA), CD68 (ready to use solution, cat #168 M-98, Cell Marque, Rocklin, CA), CD163 (ready to use solution, cat #163 M-18, Cell Marque, Rocklin, CA), and Iba1 (1:100 dilution, cat#PA5-27436, Invitrogen, Carlsbad, CA); diaminobenzidene (DAB) was used as chromogen with hematoxylin counter stain. Examples of the immunohistochemical stains for the microglial markers are presented in Fig. 1. Images were acquired for analysis using a high-speed, high-resolution Olympus VS110 virtual slide scanning system and VS-ASW software (Olympus America Inc., Center Valley, PA) at 10X magnification.

**Fig. 2:**
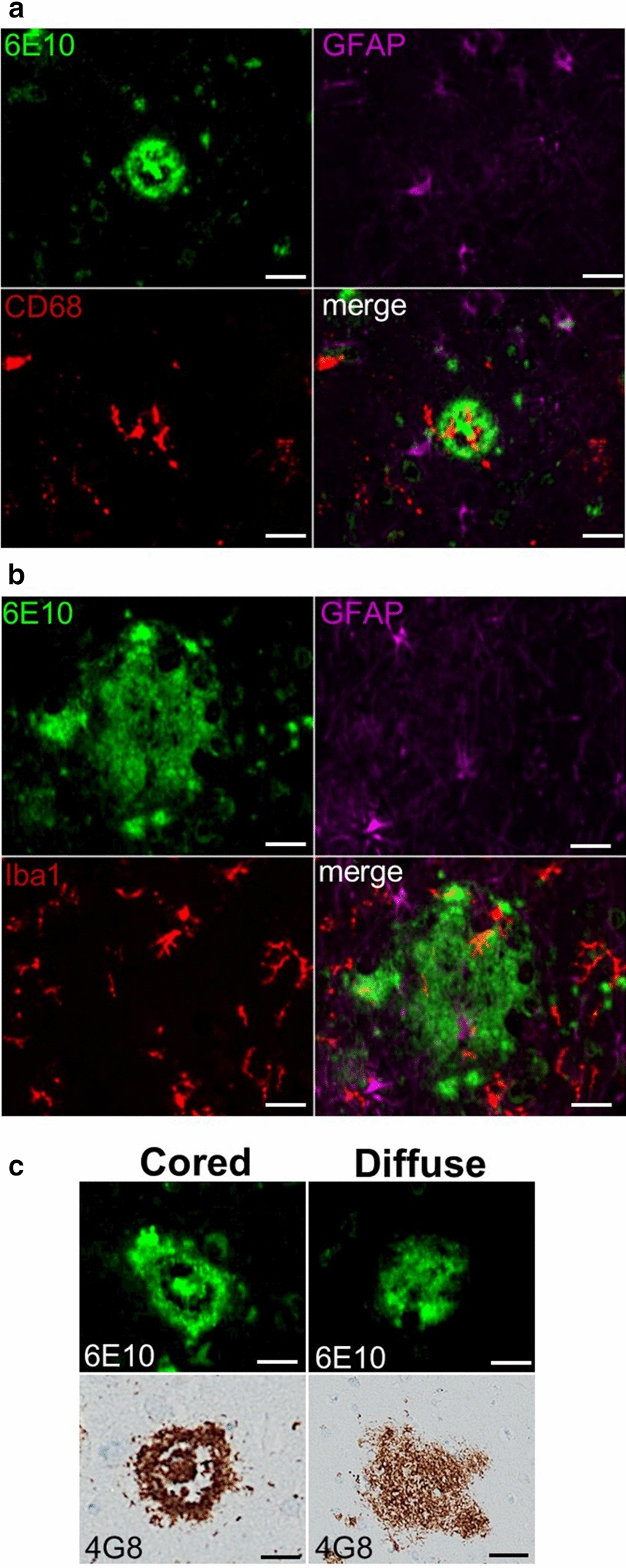
Representative multiplex immunofluorescence images of cored (2a) and diffuse (2b) plaques, and correlations between fluorescence and immunohistochemical staining of A$\upbeta$ with antibodies 6E10 and 4G8 (2c). Scale bar 20um for all images. **a** multiplex immunofluorescence of a cored plaque stained for Aβ (6E10 primary antibody with AF488 secondary antibody, green), GFAP (AF555 secondary antibody, magenta), CD68 (AF647 secondary antibody, red); with merged channels in the bottom right of the panel. **b** multiplex immunofluorescence of a diffuse plaque stained for Aβ (6E10 primary antibody with AF488 secondary antibody, green), GFAP (AF555 secondary antibody, magenta), and Iba1 (AF647 secondary antibody, red) with merged channels in the bottom right of the panel. 2c: Examples of cored and diffuse Aβ plaques detected by immunofluorescence with primary antibody 6E10 (row 1) and diaminobenzidene immunohistochemistry with primary antibody 4G8 (row 2)

A subset of 31 brains were selected for higher power, multiplex immunofluorescence (IF) analysis on the basis of having parenchymal A$\upbeta$ deposition with a minimum of 19 discrete plaques available for analysis, and age-matching of HIV-infected and HIV-neg decedents. Staining for A$\upbeta$, GFAP, and either Iba1 or CD68 on serial sections was performed. Primary antibodies and antisera to detect A$\upbeta$ (mouse clone 6E10, 1:1000 dilution, cat#803001, Biolegend, San Diego, CA), GFAP (rat clone 2.2B10, 1:500 dilution, cat#130300, Invitogen, Carlsbad, CA), and either CD68 (rabbit clone SP251, 1:50 dilution, cat#SAB5500070, Sigma-Aldrich**,** St. Louis, MO) or Iba1 (rabbit antiserum, 1:100 dilution, cat#PAS27436, ThermoFisher, Carlsbad, CA) were utilized with secondary reagents Alexa Fluor (AF) 488 donkey anti-mouse (as supplied, cat#A21202), AF555 donkey anti-rat (as supplied, cat#48269), and AF647 donkey anti-rabbit (as supplied, cat#A31573), and nuclear counterstaining with Hoechst 33342 trihydrochloride trihydrate (cat#H3570, Invitrogen, Carlsbad, CA). Amyloid detection by 6E10 (in contrast to 4G8) was chosen for this analysis as it provided clearer images of A$\upbeta$ plaques that could be delineated under fluorescence, as 6E10 does not react with intracellular glial A$\upbeta$ granules, and therefore allows a clearer delineation of extracellular A$\upbeta$ plaque [25]. Images were acquired for analysis using a high speed, high resolution Nano Zoomer S60 scanner (Hamamatsu Corporation, Japan) at 20 × magnification, available through the ISMMS Department of Oncological Sciences Histopathology Core Facility (see Fig. 2 for examples of IF staining).

### Scoring for neurodegenerative proteins

**Fig. 3:**
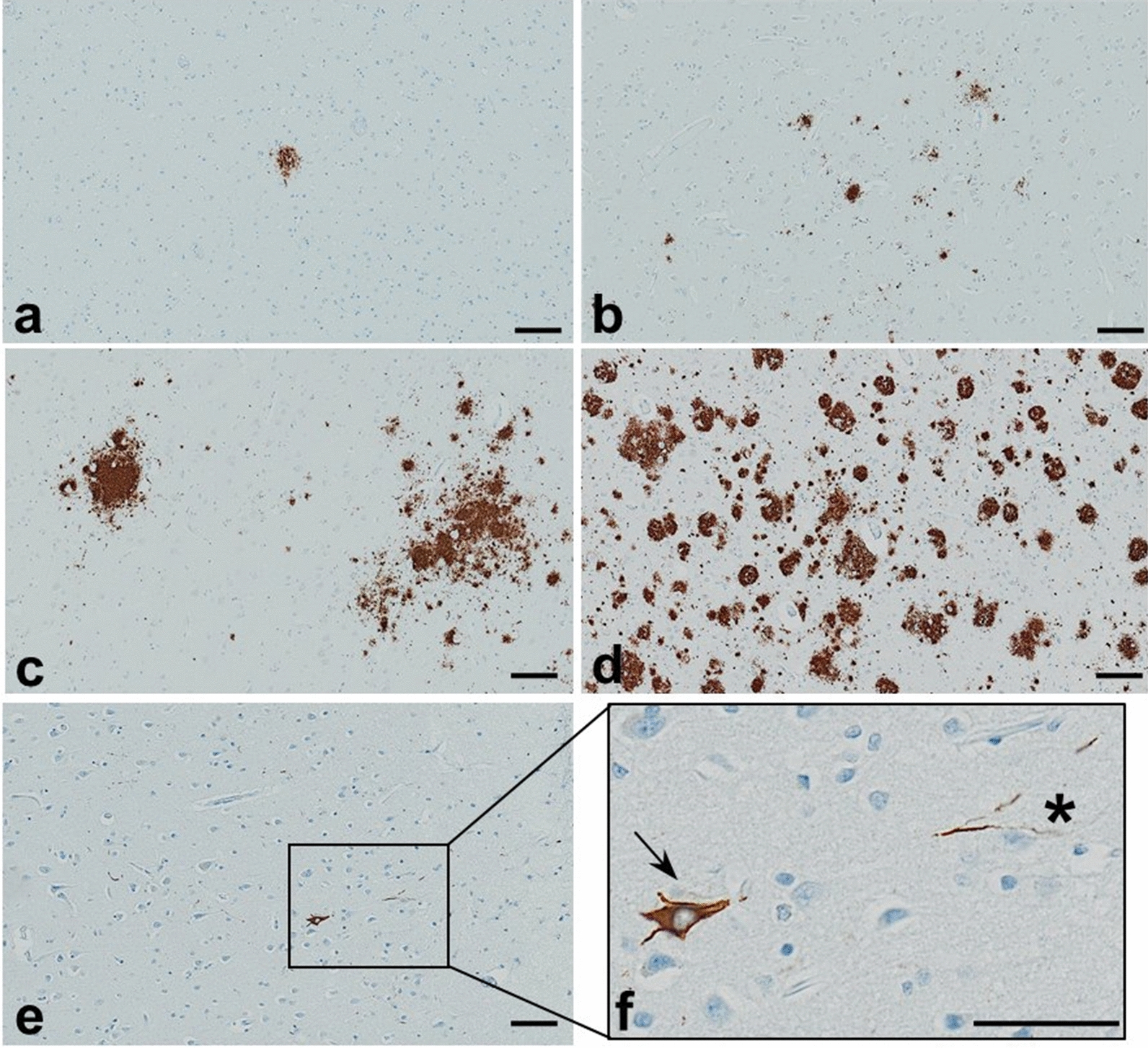
Scoring of Aβ plaques in frontal cortex by immunohistochemistry with antibody 4G8 (**a**-**d**), and representative images of low density, unscored p-tau pathology detected by immunohistochemistry with antibody AT8 (**e**–**f**). Severity ratings of frontal Aβ are illustrated: (**a**) rare: isolated plaque (score 0.5); (**b**) mild: more than 3 plaques in a single region (score 1); (**c**) moderate: multiple plaques in multiple discontinuous regions of cortex (score 2); and (**d**) severe: numerous plaques confluent throughout the cortical ribbon (score 3). Panels (**e**, **f**) demonstrate p-tau pathology; a neuronal tangle can be seen in low power (**e**), and the region outlined by a rectangle is shown in higher power in (**f**), with an isolated neurofibrillary tangle indicated by an arrow, and threads indicated by an asterisk. Panels (**a**-**e**) scale bar 100um, panel (**f**) scale bar 50um

The presence or absence of parenchymal A$\upbeta$ deposition and p-tau accumulations in neuronal perikarya was assessed independently by two diagnostic neuropathologists (SM, EPC) examining DAB-IHC slides by light microscopy, and finalized through consensus. Neuronal p-tau was scored as present or absent. For A$\upbeta$ plaques, in addition to coding for their presence or absence, the severity of frontal deposition was scored on an ordinal scale: 0 (no plaques identified); 0.5 (rare; fewer than 3 small plaques); 1 (mild; 3 or more small plaques or 1 or more large lakes of A$\upbeta$ in a single region of cortex); 2 (moderate numbers of large or small plaques in multiple discontinuous regions of cortex); 3 (numerous plaques extending confluently throughout cortex). Examples of these stages are presented in Fig. 3, along with a representative example of frontal neuronal p-tau. In 242 individuals these frontal severity scores could be correlated with medial temporal lobe stages of amyloid deposition, as described by Thal and colleagues [26]. To further validate the visual amyloid scoring of sections by the 2 neuropathologists, their ratings were correlated with quantitative QuPath analysis (see “Image Analysis” section below); sections called with parenchymal A$\upbeta$ by the neuropathologists had a significantly higher median value for the percent area occupied by A$\upbeta$ than those without (after log transformation median [IQR} with plaques 0.058 [0.020, 0.364], without plaques, 0.007 [0.004, 0.014], *p* < 0.0001). However, QuPath measures did not distinguish between parenchymal and intracellular amyloid, and were therefore not utilized in analysis. For higher power IF analyses, discrete plaques were outlined manually and their areas measured in QuPath, as described below.

### Image analysis

All acquired images were analyzed with QuPath version 0.2.1, downloaded from the homepage located at Github (https://QuPath.github.io/) [27]. Regions of interest (ROI) for DAB IHC were identified initially on the section stained for A$\upbeta$, and included: a large, 44.797 mm^2^ polygon encompassing the cortical ribbon; a 14.366 mm^2^ polygon in underlying cerebral white matter; and two, smaller 5.052 mm^2^ cortical ellipses which for cases with parenchymal amyloid deposition were chosen with one identifying a region containing amyloid plaques and a second in a region without plaque. If no plaques were present, only one ellipse without amyloid was utilized; in 22 cases, plaques were extensive throughout the cortical ribbon, and only one ellipse with amyloid was analyzed. Scripts were created to extract/copy ROI annotations from the initial A$\upbeta$ image, and these annotations were superimposed on the three corresponding images of microglial markers (CD68, CD163, Iba1) for each case. Transfers were checked manually and rotated if necessary (adjusting for positioning of sections on the slides). Once ROIs were correctly placed, the area occupied by DAB was measured with the pixel classification command and standardized thresholds in the DAB channel*.* Each image was manually checked post classification, and annotation measurements were used to calculate the percentage areas of each microglial marker.

QuPath was also used for analysis of glial cells in the immediate microenvironment of A$\upbeta$ plaques. To center this analysis, plaques were outlined by a pixel classifier based upon an intensity threshold applied to the AF488 A$\upbeta$ channel, and objects with a minimum area of 280 um^2^, to reflect a minimum diameter of approximately 20 um, were manually selected. This area threshold was used to exclude isolated “stellate” deposits not clearly in the extra-cellular space [28, 29]. Based on morphology, plaques were characterized as dense core or diffuse (Fig. 2); diffuse were more numerous than cored, and 7 patients did not display appreciable cored plaques. Between 18 and 25 diffuse and 8 and 13 cored plaques per patient were analyzed. To define the immediate plaque microenvironment, an area surrounding each labeled plaque with a radius of 50um was drawn by using the QuPath “expand annotations” command. Thus, the regional area occupied by glia could be assessed both within each continguous amyloid deposit and in the immediately adjacent neuropil (Additional file 1: supplemental Fig. 1).

### Genotyping

APOE genotyping was performed as previously described, using Taqman Assays-on-demand (Applied Biosystems, Foster City, CA) which target rs429358 and rs7412 [22].

### Statistical procedures

Groups were defined by virologic status prior to death. Between group comparisons were conducted with analysis of variance, Kruskal–Wallis/Wilcoxon, or $\upchi$-squares tests. Tests of relatedness between both predictor and outcome variables were done through Spearman rank order correlations. Microglial markers were modeled continuously. For DAB IHC, the four ROIs (cortical ribbon, sub-regions of amyloid-containing and amyloid-free cortex, and white matter) were examined, as well as a normally-distributed, within-individual variable reflecting the difference between amyloid-containing and amyloid-free regions (translated + 1 to avoid negative numbers). For IF analyses an average value for each plaque type per patient was computed prior to between group analyses; to improve skewness, percentage areas were log transformed. Paired t tests were used to examine differences in microglial markers between plaque centers and rims. Logistic regression was used to construct models to predict p-tau and A$\upbeta$, with minimization of Bayesian Information Criterion (BIC) used to optimize forward stepwise selection of predictors. Models were also created to predict microglial populations through similar stepwise selection. All analyses were generated using JMP version 9.0 for Macintosh (SAS Institute Inc., Cary, NC).

## Results

### Patient population, and distribution of p-tau and A$\upbeta$ pathologies

Characteristics of the sample are displayed in Table 1. The mean age of the overall sample was 52.8 ± 0.75 years. The HIV-D group died at a significantly younger age (47.7 years) than both HIV-U (58.2 years) and HIV-neg (53.3 years). As the youngest group, HIV-D had less hypertension (HTN) and diabetes mellitus (DM) than HIV-U and HIV-neg. As expected, the HIV-D group had significantly lower CD4 T-cell counts and shorter duration HIV disease than HIV-U.

**Table 1:** Clinical characteristics of the study population

	Total sample (n = 254)	HIV negative (HIV-neg) (n = 63)	HIV positive – undetectable** (HIV-U) (n = 91)	HIV positive – detectable (HIV-D) (n = 100)	p
Mean age (SEM)^a^	52.8 (0.75)	53.3 (1.4)	58.2 (1.2)	47.7 (1.1)	< 0.0001
Male sex, n (%)	157 (61.8%)	39 (61.9%)	57 (62.6%)	61 (61.0%)	0.9732
Race/ethnicity, n (%) Black Hispanic White Other	113 (44.5%) 85 (33.5%) 49 (19.3%) 7 (2.7%)	27 (42.9%) 22 (34.9%) 12 (19.1%) 2 (3.1%)	36 (39.6%) 30 (33.0%) 23 (25.2%) 2 (2.2%)	50 (50.0%) 33 (33.0%) 14 (14.0%) 3 (3.0%)	0.5991
APOE $\upvarepsilon$4, n (%)*^b^	76 (30.0%)	18 (28.6%)	20 (22.0%)	38 (38.4%)	0.0460
APOE $\upvarepsilon$2, n (%)*	47 (18.6%)	11 (17.5%)	17 (18.7%)	19 (19.2%)	0.9621
Median CD4 count cells/mm3 [IQR]^c^	NA	NA	278 [133,503]	74 [12,187]	< 0.0001
Median log plasma HIV load [IQR]^d^	NA	NA	1.69 [1.69,1.69]	4.67 [2.80,5.53]	< 0.0001
Mean duration of HIV disease in years (SEM)^c^	NA	NA	18.6 (0.8)	12.7 (0.7)	< 0.0001
Frontal parenchymal amyloid deposition, n (%)^e^	78 (30.7%)	25 (39.7%)	34 (37.4%)	19 (19.0%)	0.0026
Moderate to severe frontal amyloid deposition, n (%)^e^	28 (11.0%)	9 (14.3%)	12 (13.2%)	7 (7.0%)	0.0120
Neuronal ptau in frontal cortex	58 (22.8%)	17 (27.0%)	22 (24.2%)	19 (19.0%)	0.4623
Hypertension^e^	118 (46.5%)	35 (55.6%)	53 (58.2%)	30 (30.0%)	0.0001
Diabetes Mellitus^f^	68 (26.8%)	26 (41.3%)	28 (30.8%)	14 (14.0%)	0.0004
Hepatitis (B or C)^g^	166 (65.4%)	31 (49.2%)	71 (78.0%)	64 (64.0%)	0.0010

^a^HIV-U > HIV-neg > HIV-D

^b^HIV-D > HIV-U, HIV-neg

^c^HIV-U > HIV-D

^d^HIV-U < HIV-D

^e^HIV-D < HIV-U, HIV-neg

^f^HIV-D < HIV-U < HIV-neg

^g^HIV-neg < HIV-D < HIV-U

^*^genotyping unavailable for 1 participant

^**^limit of detection: 49 copies HIV RNA/ml plasma (log value 1.69)

SEM: standard error of the mean; IQR: interquartile range

Frontal neuronal p-tau was identified in 58 (22.8%), and frontal A$\upbeta$ plaques in 78 (30.7%) individuals. These two pathologies were significantly correlated; 27 individuals had p-tau in neurons and A$\upbeta$ plaques, 31 had p-tau in neurons without A$\upbeta$ plaques, 51 had A$\upbeta$ plaques and no neuronal p-tau, and 145 had neither neuronal p-tau nor frontal plaques; 34.6% of those with A$\upbeta$ had neuronal p-tau, in contrast to 17.6% of those without A$\upbeta$ (*p* = 0.0029, $\upchi$2 analysis).

A greater proportion of individuals in the HIV-D group had an APOE $\upvarepsilon$4 allele (38.4%) compared to HIV-U (22.0%) and HIV-neg (28.6%). Despite this, the younger HIV-D group had significantly fewer individuals with A$\upbeta$ plaques than the other groups. The three groups had equivalent proportions with p-tau in frontal neuronal perikarya. P-tau pathologies were not scored for severity, as only 3 decedents had numerous frontal tangles, pre-tangles and threads; the remainder of individuals displaying frontal p-tau neuronal immunoreactivity had low density pathology. Frontal A$\upbeta$ plaques were assessed for severity, and were rare in 22 (8.7%), mild in 28 (11.0%), moderate in 12 (4.7%), and severe in 16 (6.3%). These severity scores correlated with medial temporal lobe phases of amyloid deposition as described by Thal and colleagues [26], and all individuals with moderate to severe frontal amyloid had medial temporal lobe amyloid, with 25 of the 28 demonstrating hippocampal/subicular distribution (equivalent to Thal medial temporal stages 3 or 4) (*p* < 0.0001). The younger HIV-D group, in addition to having a smaller proportion of individuals with frontal A$\upbeta$, also had a smaller percentage with moderate to severe frontal plaque pathology.

### Bivariate correlations of microglial cell populations with HIV status, neurodegenerative proteins, cART formulations, and co-morbid diseases

**Fig. 4:**
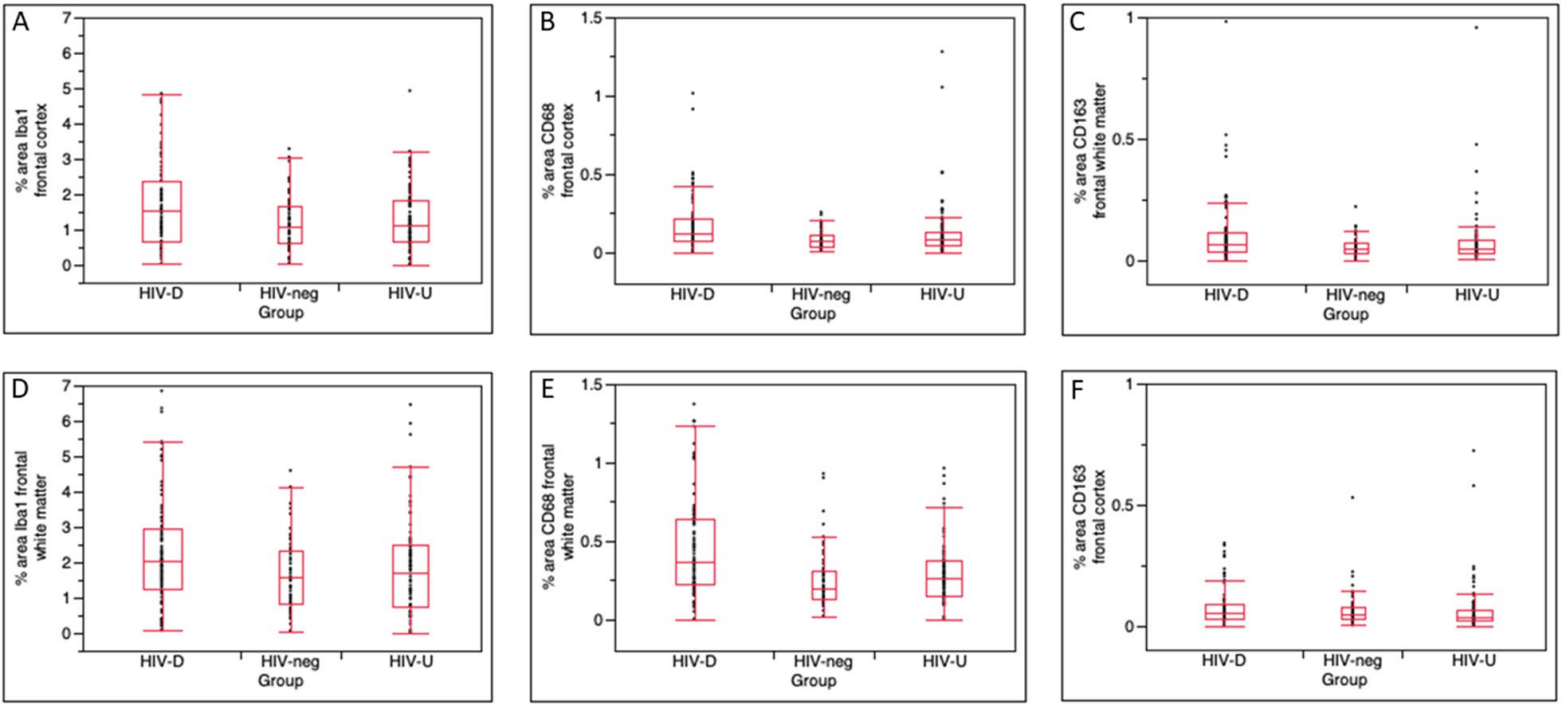
Box and whiskers plots to demonstrate medians and interquartile ranges of staining for microglial markers in HIV-negative (HIV-neg), HIV-positive with detectable viral load (HIV-D) and HIV-positive with undetectable viral load (HIV-U) patient groups. Top row demonstrates measures in frontal cortex, with percentage areas occupied by staining for Iba1(**a**), CD68 (**b**), and CD163 (**c**); bottom row demonstrates measures in frontal white matter, with percentage areas occupied by staining for Iba1(**d**), CD68 (**e**), and CD163 (**f**). For all markers, HIV-D had significantly greater median staining than the other groups (see Table 2)

As anticipated, unsuppressed HIV disease had a significant impact on microglial cell populations (Table 2 and supplemental Table 1). In general, for all markers (Iba1, CD68, CD163), HIV-D had greater expression in frontal gray and white matter than other groups (Table 2 and Fig. 4); for CD68, this difference was also significant in smaller areas measured with and without parenchymal A$\upbeta$ (supplemental Table 1). Across the entire sample, there was no relationship between the area occupied by microglial cell markers Iba1, CD68, or CD163 in cortex and deposition of A$\upbeta$ or presence of p-tau containing neurons (all bivariate analyses p > 0.0500; Table 3). There was similarly no correlation of these markers with frontal A$\upbeta$ severity scores. For 56 of the 78 individuals with cortical amyloid deposition, a variable reflecting the difference in microglial cell markers in regions with and without plaque was constructed; the mean within-individual difference was the same for all 3 groups (supplemental Table 1). When HIV-D were excluded from analyses, as with the entire sample, no significant relationship could be discerned between the presence of neurodegenerative proteins and area occupied by activated or total microglial cells.

**Table 2:** Frontal lobe microglial cell percentage areas in HIV-negative and HIV-positive individuals with and without control of plasma viremia

	Total sample n = 254	HIV-neg n = 63	HIV-U n = 91	HIV-D n = 100	p
CD68 cortex^a^	0.0953 [0.0509,0.1646]	0.0699 [0.0361,0.1146	0.0820 [0.0490,0.1275]	0.1200 [0.0748,0.2176]	< 0.0001
CD163 cortex^b^	0.0442 [0.0261,0.0767]	0.0484 [0.0266,0.0762]	0.0348 [0.0202,0.0670]	0.0537 [0.02768,0.0919]	0.0551
Iba1 cortex^a^	1.1979 [0.6588,1.9662]	1.0879 [0.6085,1.6644]	1.1252 [0.6600,1.8503]	1.5518 [0.6813,2.3906]	0.0154
CD68 WM^a^	0.2739 [0.1562,0.4391]	0.1967 [0.1261,0.3117]	0.2619 [0.1450,0.3748]	0.3709 [0.2230,0.6377]	< 0.0001
CD163 WM^a^	0.0500 [0.0306,0.0897]	0.0459 [0.0292,0.0687]	0.0463 [0.0267,0.0817]	0.0655 [0.0342,0.1160]	0.0097
Iba1 WM^a^	1.8098 [0.9741,2.5976]	1.5831 [0.8477,2.3285]	1.6913 [0.7707,2.4884]	2.0447 [1.2424,2.9518]	0.0214

HIV-neg: HIV negative; HIV-U: HIV undetectable; HIV-D: HIV detectable; WM: subcortical white matter; A$\upbeta$: Amyloid beta

Median and inter quartile range displayed for all markers

P values refer to comparisons between HIV-neg, HIV-U, and HIV-D groups by Kruskal–Wallis tests

^a^HIV-D > HIV-U, HIV-neg ^b^HIV-D > HIV-U

**Table 3:** Cortical microglial percentage areas with and without co-morbid diseases and neurodegenerative protein accumulations

Full sample (n = 254)	CD68			CD163			Iba1		
	With condition	Without condition	p	With condition	Without condition	p	With condition	Without condition	p
HTN	0.0751 [0.0389,0.1278]	0.1059 [0.0603,0.1799]	**0.0017**	0.0361 [0.0222,0.0680]	0.0535 [0.0279,0.0877]	**0.0212**	1.0889 [0.6399,1.8770]	1.3454 [0.6705,2.0336]	0.1165
Diabetes	0.0761 [0.0386,0.1236]	0.1045 [0.0542,0.1766]	**0.0109**	0.0426 [0.0275,0.0811]	0.0454 [0.0243,0.0767]	0.6317	0.9892 [0.5738,1.5882]	1.3648 [0.6805,2.0385]	**0.0054**
Hepatitis	0.0928 [0.0538,0.1555]	0.0958 [0.0400,0.1689]	0.5971	0.0420 [0.0250,0.0767]	0.0484 [0.0273,0.0780]	0.4024	1.2183 [0.6609,2.0385]	1.1696 [0.6526,1.8135]	0.4450
APOE $\upvarepsilon$4	0.1073 [0.0554,0.1775]	0.0861 [0.0497,0.1638]	0.3147	0.0512 [0.0262,0.0822]	0.0420 [0.0255,0.0750]	0.3912	1.1969 [0.6358,2.0196]	1.1966 [0.6683,1.9456]	0.9813
A$\upbeta$ plaques	0.0820 [0.0392,0.1565]	0.0983 [0.0566,0.1715]	0.0956	0.0470 [0.0294,0.0775]	0.0420 [0.0238,0.0764]	0.6610	1.1041 [0.6575,1.7180]	1.2220 [0.6566,2.0110]	0.3744
Neuronal p-tau	0.1022 [0.0480,0.1979]	0.0912 [0.0512,0.1564]	0.3734	0.0447 [0.0258,0.0958]	0.0442 [0.0260,0.0734]	0.6174	1.2106 [0.7226,2.3159]	1.1979 [0.6431,1.9511]	0.6318
sub-sample excluding HIV-D (n = 154)									
HTN	0.0716 [0.0353,0.1203]	0.0871 [0.0487,0.1478]	0.1582	0.0358 [0.0228,0.0700]	0.0484 [0.0244,0.0706]	0.6521	1.0889 [0.6383,1.8554]	1.1207 [0.6275,1.7292]	0.8082
Diabetes	0.0736 [0.0377,0.1258]	0.0825 [0.0475,0.1258]	0.5895	0.0414 [0.0277,0.0778]	0.0356 [0.0204,0.0677]	0.1392	0.9165 [0.5469,1.4358]	1.2040 [0.6767,1.8008]	0.0601

Values displayed are median and inter quartile range. P values for differences in microglial areas between groups with and without conditions listed in column one; significant values by Kruskal–Wallis/Wilcoxon tests bolded. HTN: hypertension

We next examined whether specific cART formulations were associated with cortical microglial cell populations. While reflecting the purpose of cART to render viral loads undetectable, the stratification of the HIV sample into HIV-U and HIV-D categories did not allow analysis of whether specific drug classes (protease inhibitors, nucleoside reverse transcriptase inhibitors, non-nucleoside reverse transcriptase inhibitors, and integrase inhibitors), or formulations of the cART backbone (emtricitabine/tenofovir, lamivudine/abacavir, or other) were associated with microgliosis. Within the treated HIV sample (n = 147), only integrase inhibitors (INSTI) were associated with decreased cortical CD68 microglia (median on INSTI 0.0551 [0.0194, 0.1167], off INSTI 0.1066 [0.0663, 0.1855], *p* = 0.0027). This was consistent with treatment effect, as individuals on INSTI had lower viral loads than those off (mean log plasma HIV on INSTI = 2.08 (0.23), off INSTI 2.85 (0.13), *p* = 0.0044). Furthermore, within the treated sample, INSTI were the only specific drug class distinguished by statistically significant associations with lower plasma HIV load. INSTI were not correlated with the presence of A$\upbeta$ [22]. Significant associations of drug classes or regimen backbones with Iba1 and CD163 were not observed.

Finally, for the overall population, there were associations between DM and HTN and cortical microglia, but not for hepatitis or APOE $\upvarepsilon$4 status (Table 3). Age was also associated with cortical CD68 (adjusted R^2^ = 0.0148, *p* = 0.0295). These associations were confounded by the younger age and lower co-morbid disease prevalence of the HIV-D group, and when HIV-D were excluded from analysis, microglial areas did not correlate with age, DM, or HTN.

### Multivariate models to predict A$\upbeta$ and p-tau

To determine whether microglia contributed to models predicting cortical presence of neurodegenerative proteins despite a lack of statistical significance in bivariate correlation, the following variables were tested with stepwise selection: age, HIV status, duration HIV (with HIV-neg coded as 0 years), percent cortical areas of CD68, CD163, and Iba1 microglia, sex, presence of an APOE $\upvarepsilon$4 allele, hypertension, and diabetes. Only age (effect likelihood ratio (LR) $\upchi$2 = 7.40, *p* = 0.0065), APOE $\upvarepsilon$4 status (LR $\upchi$2 = 11.61, *p* = 0.0007), HIV duration (LR $\upchi$2 = 9.66, *p* = 0.0019), and HIV status (LR $\upchi$2 = 12.56, *p* = 0.0004) were retained in a significant nominal logistic regression to predict parenchymal amyloid (R^2^ = 0.1420, *p < *0.0001). When the same predictors were tested in a model of frontal neuronal p-tau, the only retained predictor was diabetes (LR $\upchi$2 = 6.03), with an R^2^ = 0.0221, *p* = 0.0141.

### Analysis of plaque-associated glial populations by immunofluorescence

The influence of HIV status on microglial cell populations is a well-documented and strong effect [17–19], which over large regions of brain may obscure local relationships with neurodegenerative protein accumulation. To examine the immediate microenvironment of A$\upbeta$ deposits, we conducted multiplex immunofluorescence to determine whether differences between the HIV groups existed both within and immediately adjacent to abnormal plaque formations, the majority of which were diffuse in conformation. As microglia acquire pro-inflammatory phenotypes in the process of aging [30], HIV-positive and negative groups were age-matched; individuals were further selected for a minimum of 19 discrete A$\upbeta$ plaques to assure adequate numbers for analysis. The resultant A$\upbeta$ plaque-bearing sub-sample was on average 10 years older than the larger autopsy population from which they were selected; characteristics of this sub-sample are displayed in Table 4.

**Table 4:** Characteristics of subsample for examination of glial cells in A$\upbeta$ plaque microenvironment, and comparison of glial cell areas between HIV-negative, HIV-undetectable, and HIV-detectable age-matched groups

	Total sample	HIV-neg	HIV-U	HIV-D	p
n	31	12	11	8	
Mean age (SEM)	61.7 (1.28)	61.7 (2.10)	62.8 (2.19)	60.1 (2.57)	0.7302
Male gender, n (%)^a^	15 (48%)	7 (58%)	2 (18%)	6 (75%)	0.0340
APOE $\upvarepsilon$4, n (%)^b^	16 (52%)	3 (25%)	6 (55%)	7 (88%)	0.0227
APOE $\upvarepsilon$2, n (%)	3 (9.7%)	2 (17%)	0	1 (13%)	0.3825
Mean area diffuse plaque, um^2^ (SEM)	2495 (310)	2213 (503)	2350 (526)	3115 (617)	0.5054
Mean area cored plaque, um^2^ (SEM)*	1560 (94)	1548 (159)	1649 (159)	1445 (194)	0.6383
cored plaques					
GFAP plaque	0.7174 [0.1098,1.2907]	0.1126 [0.0305,1.2316]	1.0813 [0.6481,1.2491]	0.2959 [0.0000,1.4296]	0.3433
GFAP rim	0.4148 [0.0736,0.9678]	0.0885 [0.0239,0.9515]	0.6775 [0.4148,0.9360]	0.2008 [0.0268,1.0748]	0.2997
CD68 plaque	0.7098 [0.1445,1.0210]	0.8035 [0.0993,1.0601]	0.8004 [0.3006,1.0100]	0.4198 [0.1282,1.2166]	0.8655
CD68 rim	0.1570 [0.6844,0.1780]	0.1632 [0.0649,0.1964]	0.1313 [0.0797,0.1766]	0.1146 [0.0494,0.2480]	0.8947
Iba1 plaque	0.9427 (0.0810)	0.9651 (0.1383)	0.9425 (0.1383)	0.9093 (0.1694)	0.9681
Iba1 rim	0.5612 (0.0504)	0.6003 (0.0845)	0.5761 (0.0845)	0.4800 (0.1034)	0.6554
diffuse plaques					
GFAP plaque	0.4327 [0.0592,0.8564]	0.1513 [0.0394,0.9381]	0.6198 [0.0969,0.7264]	0.3205 [0.1084,0.8807]	0.4226
GFAP rim	0.3135 [0.0558,0.6165]	0.1392 [0.0509,0.6278]	0.4609 [0.0781,0.6165]	0.3612 [0.0763,0.7271]	0.5281
CD68 plaque	0.1682 [0.0931,0.3177]	0.1478 [0.1027,0.2158]	0.1970 [0.0641,0.3547]	0.2592 [0.0664,0.3815]	0.6105
CD68 rim	0.1492 [0.1099,0.1673]	0.1444 [0.1097,0.1937]	0.1489 [0.1086,0.1673]	0.1576 [0.1257,0.2428]	0.5010
Iba1 plaque	0.7593 (0.0602)	0.7855 (0.0994)	0.7077 (0.1038)	0.7906 (0.1217)	0.8269
Iba1 rim	0.5970 (0.0452)	0.6353 (0.0745)	0.5556 (0.0779)	0.5965 (0.0913)	0.7626

Plaque areas and percentage glial cell areas log transformed prior to analysis; for GFAP and CD68 medians and IQR, for Iba1, means and SEM. *: cored plaques not sufficient in numbers for analysis in 2 HIV-D, 2 HIV-U, and 3 HIV-neg a: HIV-U < HIVD, HIV-neg; b: HIV-D > HIV-U > HIV-neg

In contrast to HIV group differences in the larger cortical areas examined by IHC, no significant HIV-associated differences in microgliosis were discerned in the immediate microenvironment of A$\upbeta$ plaques (Table 4). However, across the entire sample, the centers of cored and diffuse plaques had greater percentage areas occupied by CD68 and Iba1-expressing microglia and GFAP-positive astrocytes when compared to the amyloid-free immediately adjacent neuropil (plaque “rims”) (paired t tests displayed in supplemental Table 2). As the age-matched IF HIV sub-groups were unbalanced with regard to APOE $\upvarepsilon$4 status and sex, we examined these variables as predictors of glial response, and then advanced HIV group status, APOE $\upvarepsilon$4 status, and sex to stepwise selection for multiple logistic regression (Table 5). In contrast to the dominant effect of HIV group in low power analysis, in the microenvironment of plaques, APOE $\upvarepsilon$4 status and sex were more frequent predictors with greater effect sizes, contributing to models of area occupied by Iba1 positive microglia in cored and diffuse plaque centers and rims; CD68 positive microglia in diffuse plaque centers; and GFAP positive astrocytes in diffuse plaque centers and both diffuse and cored plaque rims. HIV group status contributed to models of Iba1 microglia in cored plaque rims and GFAP in diffuse plaque centers and rims, but had smaller effect size than both APOE $\upvarepsilon$4 and sex in these models. The effect of APOE $\upvarepsilon$4 and sex was such that individuals who were male or had an $\upvarepsilon$4 allele displayed greater percentage areas occupied by glia in the A$\upbeta$ plaque microenvironment.

**Table 5:** Predictors of percentage glial area in the immediate A$\upbeta$ plaque microenvironment

	APOE $\upvarepsilon$4 positive	APOE $\upvarepsilon$4 negative	p	Male sex	Female sex	p	Multivariate analysis	
	n = 16	n = 15		n = 15	n = 16		adjusted r2, p	predictor F ratio, p
Cored plaques								
GFAP plaque	1.1153 [0.2096,1.4996]	0.6259 [0.0216,1.0250]	0.0770	1.0167 [0.0434,1.3705]	0.6481 [0.1276,1.1448]	0.7288	no significant models	
GFAP rim	0.8644 [0.1481,1.2017]	0.0885 [0.0128,0.6391]	0.0162	0.4805 [0.0457,1.2213]	0.4148 [0.0736,0.7320]	0.5254	adj r2 = 0.1748 *p* = 0.0241	APOE $\upvarepsilon$4 F = 5.8712 *p* = 0.0241
CD68 plaque	0.8004 [0.1787,1.1041]	0.5310 [0.1028,0.9554]	0.4009	0.7098 [0.1445,1.0485]	0.7086 [0.1428,1.0210]	0.9081	no significant models	
CD68 rim	0.1313 [0.0709,0.2012]	0.1620 [0.0615,0.1708]	0.9307	0.1632 [0.0642,0.2460]	0.1416 [0.0689,0.1706]	0.6439	no significant models	
Iba1 plaque	1.0879 (0.1029)	0.7711 (0.1119)	0.0049	1.0833 (0.1093)	0.8021 (0.1093)	0.0824	adj r2 = 0.1270 *p* = 0.0489	APOE $\upvarepsilon$4 F = 4.3449 *p* = 0.0489
Iba1 rim	0.6145 (0.0680)	0.4982 (0.0739)	0.2595	0.6496 (0.0679)	0.4728 (0.0679)	0.0790	adj r2 = 0.1950 *p* = 0.0395	sex F = 6.4975 *p* = 0.0187 group (HIV-D) F = 3.7534 *p* = 0.0663
diffuse plaques								
GFAP plaque	0.6471 [0.2113,1.0093]	0.0592 [0.0382,0.6198]	0.0057	0.6681 [0.0948,1.0842]	0.1733 [0.0485,0.6322]	0.0820	adj r2 = 0.3369 *p* = 0.0049	APOE $\upvarepsilon$4 F = 8.1618 *p* = 0.0083 sex F = 7.3948 *p* = 0.0115 group (HIV-D) F = 3.2991 *p* = 0.0529
GFAP rim	0.5597 [0.1759,0.8201]	0.1045 [0.0492,0.4170]	0.0143	0.4459 [0.0558,0.8549]	0.2105 [0.0573,0.5591]	0.1331	adj r2 = 0.3074 *p* = 0.0047	APOE $\upvarepsilon$4 F = 5.7802 *p* = 0.0233 sex F = 6.3260 *p* = 0.0182 group (HIV-U) F = 3.9262 *p* = 0.0578
CD68 plaque	0.3024 [0.1512,0.3865]	0.1065 [0.0358,0.1749]	0.0072	0.1749 [0.1065,0.2993]	0.1555 [0.0778,0.3772]	0.8433	adj r2 = 0.1081 *p* = 0.0397	APOE $\upvarepsilon$4 F = 4.6366 *p* = 0.0397
CD68 rim	0.1565 [0.1110,0.2460]	0.1356 [0.1028,0.1605]	0.1989	0.1504 [0.1086,0.1667]	0.1442 [0.1110,0.2108]	0.9527	no significant models	
Iba1 plaque	0.8684 (0.0800)	0.6428 (0.0826)	0.0595	0.9031 (0.0797)	0.6244 (0.0772)	0.0178	adj r2 = 0.1504 *p* = 0.0178	sex F = 6.3117 *p* = 0.0178
Iba1 rim	0.6526 (0.0623)	0.5377 (0.0643)	0.2095	0.6865 (0.0620)	0.5131 (0.0600)	0.0537	adj r2 = 0.0921 *p* = 0.0537	sex F = 4.0449 *p* = 0.0537

All percentage glial areas log transformed prior to analysis; median and IQR for measures of CD68 and GFAP, mean and SEM for measures of Iba1. GFAP: glial fibrillary acidic protein; adj r2 = adjusted r2; plaque measurements taken within region of A$\upbeta$ deposition; rim measurements obtained within 50 um radius from the plaque border. Multivariable models included HIV group status as a potential predictor (bivariate analysis for this predictor is displayed in supplemental Table 1)

### Association of microglial cell markers and cognitive status in PWH

**Fig. 5:**
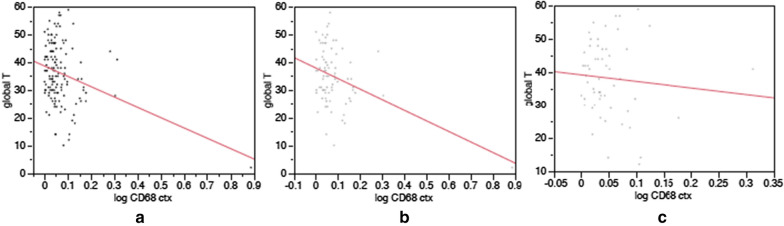
Scatterplots of global T scores by percent cortical area of CD68-expressing microglia for entire cognitively characterized HIV sample (**a**), the HIV-D subgroup (**b**), and the HIV-U subgroup (**c**). Correlations of microglial area and test performance were significant for the entire sample and the HIV-D subgroup, but not HIV-U (CD68 area log transformed prior to correlation; adjusted R2 for entire sample: 0.0887, *p* = 0.0002, for HIV-D: 0.1481, *p* = 0.0003, for HIV-U -0.0107, *p* = 0.5218)

Global T scores were examined as an index of cognitive function in 135 PWH; the overall sample was mildly impaired prior to death, with a mean score of 36.2 (0.9 SEM). Global T scores were significantly correlated with large cortical areas of microglia expressing CD68 (adjusted R^2^ = 0.0887, *p* = 0.0002), CD163 (adjusted R^2^ = 0.0637, *p* = 0.0017), and Iba1 (adjusted R^2^ = 0.0300, *p* = 0.0240), as ascertained by DAB IHC (Fig. 5). When the HIV population was split by virologic status, significant correlations were strengthened in HIV-D, but lost in HIV-U (for HIV-U, all p > 0.0500). For HIV-D, coefficients with global T scores were: for CD68 adjusted R^2^ = 0.1481, *p* = 0.0003; for CD163 adjusted R^2^ = 0.1183, *p* = 0.0011; and for Iba1 adjusted R^2^ = 0.0846, *p* = 0.0054. These effects were driven by the small fraction of measurements with extreme microgliosis; when censored, no significant correlations were observed. Finally, analysis of cognitive domains revealed significant correlation of motor function with CD68, CD163, and Iba1; learning (memory encoding) with CD68 and CD163; abstraction/executive functioning with CD68; and marginal correlation of verbal fluency with CD68 (supplemental Table 3).

## Discussion

The relationship of microglial cells to A$\upbeta$ and p-tau accumulation in human brain has been a focus of investigation for several decades, and with rare exception, has been examined in the context of aging and AD neuropathology [2, 3, 5, 6, 8, 31–33]. Microglia expressing CD68, CD163, and Iba1 have been quantitated, with many studies using similar methodologies to those employed in studies of HIV-associated microgliosis [2, 11, 17–19]. While a well-developed literature yielding insights into AD pathogenesis has evolved, there is still a relative paucity of information with regard to whether brain microglia play a role in the initiation of abnormal mid-life protein deposition, and whether co-morbid, non-AD disorders influence the relationship of neuroinflammation to neurodegeneration. Questions of mid-life initiation are particularly difficult to examine; if as hypothesized, abnormal proteostasis begins 20 years prior to clinical manifestations, autopsy cohorts of individuals in their 40s and 50s would need to be interrogated to evaluate the earliest changes relevant to AD [20]. In addition to the decreased likelihood of sizeable autopsy cohorts with early demise, analysis would be complicated by the low frequency of identifiable lesions. In a sample of 95 brains from decedents aged 40 to 50 at the Medical Examiner’s Office in Baltimore, MD, A$\upbeta$ plaques were present in only 14% [34]. In the largest study of over 2300 brains obtained in European university hospitals, 10.6% ages 41–50 and 24.5% ages 51–60 had A$\upbeta$ deposition [35]. The nascence of A$\upbeta$ plaques in middle-age also underscores the need for the sensitivity of histologic observation: the normal age-specific frequency of amyloid detection by PET-ligand neuroimaging is 0% between ages 50–59 [36].

Autopsy cohorts of PWH provide a unique opportunity to study early events in AD pathogenesis, and to examine how a disease that modulates immunity impacts the relationship between microglia and neurodegenerative protein deposition. Active systemic HIV infection results in CNS inflammation and microgliosis regardless of brain viral replication, and cART is a potent disease modifier, rendering virus undetectable and reducing systemic and neuro-inflammation [17, 21, 37]. With uncontrolled viremia or delay in cART initiation, there is immune dysregulation and the life span is shortened [38]. This is reflected in our cohort; the average age at death for individuals with active viral replication (HIV-D) was on average 5 to 10 years shorter than the other groups. In mid-frontal cortex, a region involved in the earliest stages of A$\upbeta$ deposition, the 19% prevalence of plaque in the HIV-D group was generally in keeping with the aforementioned Baltimore Medical Examiner study [34, 39]. Unexpectedly, the prevalence of APOE $\upvarepsilon$4 in the HIV-D group was significantly greater than the older HIV-U and HIV-neg groups; there is a controversial literature that suggests enhanced HIV progression and earlier death occur in individuals who possess APOE $\upvarepsilon$4 alleles [40–42]. Regardless of effects on mortality, despite the increased APOE $\upvarepsilon$4 frequency, and with significantly greater brain inflammation, the HIV-D group had roughly half the frequency of A$\upbeta$ plaques compared with HIV-U and HIV-neg. This suggests that in spite of an enhanced genetic risk, the presence of significantly greater CD68, CD163, and Iba1 expressing microglia does not initiate increased deposition of A$\upbeta$, and that other factors related to abnormal senescence might be more important. As we confirm herein with multivariable modeling, the stressors summated by HIV disease duration and other traditional AD risk factors (age and APOE $\upvarepsilon$4), but not the extent of microgliosis, appear relevant to initiation of amyloid deposits [22].

Whereas we previously hypothesized that the effect of HIV-duration on A$\upbeta$ risk might be due to prolonged exposure to pro-inflammatory stimulus, it was suggested that other HIV-associated factors, such as the duration or type of potentially neurotoxic cART regimens, might be equally suspect [43]. Summation of lifetime cART exposures at the time of autopsy is not straightforward, as accurate recording of decades of exposure and compliance is typically not feasible. In another brain tissue-based human study that analyzed the last cART regimen prior to death, tenofovir exposure decreased the odds of frontal A$\upbeta$ deposition [44]. In another study of cerebrospinal fluid (CSF) biomarkers in 70 PWH, ART exposure, and in particular protease inhibitor use, was associated with higher levels of A$\upbeta$1–42 and A$\upbeta$1–40 [45]. Taken together, these studies may suggest protective effects of cART with regard to A$\upbeta$ plaque formation, but this is controversial. In a more recent analysis of CSF biomarkers in 329 PWH, no associations between cART formulations and AD-like patterns of A$\upbeta$1–42, tau, and p-tau were identified [46]. It has also been suggested that cART exposures impact microglial cell function and the ability to process neurodegenerative proteins, however, this evidence is largely limited to cell culture systems and animal models, as in human study it might be difficult to dissect an effect of therapy on microglial cell biology above and beyond that actuated by viral control [47–50]. This is reflected in our analysis; while INSTI were associated with lower levels of activated CD68-positive microglia, this was paralleled by their ameliorative impacts on viral load; in prior work with this cohort, we saw no relationship between INSTI-containing cART regimens and cortical deposition of A$\upbeta$ [22]. In light of the previous literature and our current findings, further analysis of other neurotoxic HIV-related risks for A$\upbeta$ are clearly warranted.

While analysis of the MHBB cohort provides no evidence for microgliosis in the initiation of amyloid deposition, it does not preclude a role for neuroinflammation in disease acceleration or promotion. The large-scale dissociation of microgliosis from A$\upbeta$ and p-tau burden in the setting of an immunomodulatory systemic disease might not be surprising, as transduction of systemic inflammatory signals across the blood–brain barrier to alter patterns of microglial activation independent of intrinsic brain pathologies is a recognized phenomenon [51]. This can be demonstrated even in advanced stages of neurodegeneration. In an autopsy study of individuals dying with late-stage AD, the presence of systemic infection modified the brain expression of microglial activation markers and soluble cytokines, albeit sometimes in counter-intuitive manners [16]. One could argue that the applicability of large scale measures of cortical inflammation to AD initiation or progression, by virtue of reflecting other disease processes that influence neuroimmunity in the absence of neurodegeneration, may be suspect. For this reason, we undertook an examination of the microenvironment surrounding A$\upbeta$ plaques, as the distribution and relationship of migratory glia to neurodegenerative proteins may demonstrate smaller scale variations not detected in measures across large regions of brain parenchyma.

Despite significant large-scale changes in microglial activation related to HIV in our cohort (Table 2), in the immediate microenvironment of both cored and diffuse A$\upbeta$ plaques these HIV- related differences were not identified (Table 4). Furthermore, in this plaque-bearing sample, APOE $\upvarepsilon$4 status and sex were significantly correlated with glial responses, and in multivariable models were more important determinants of plaque-associated gliosis than HIV (Table 5). The influence of APOE status on A$\upbeta$- and p-tau-associated microgliosis has been observed in animal models, morphologic human brain studies, and by single-nucleus RNA-sequencing (snRNA-seq) analysis of microglial populations in AD human frontal cortex [3, 5, 31–33, 52, 53]. Studies of amyloid- or AD-associated neuroinflammation have generally supported a pro-inflammatory role for APOE $\upvarepsilon$4, with enhanced secretion of cytokines such as IL-6, IL-1$\upbeta$ and TNF$\upalpha$, greater in vivo counts of activated microglia, enhanced expression of CD68 and HLA-DR particularly in association with diffuse plaques, and increased density of Iba1-positive cells with p-tau lesions [3, 5, 31–33, 52]. However, these observations are not universal, and it has been pointed out that with regard to plaque-associated microglia, the timing of the observation with regard to the lifespan, temporal aspects of disease, and the spatial proximity to A$\upbeta$ deposition, are important in defining microglial cell phenotypes [54]. In one study utilizing laser-capture microdissection to isolate plaque-associated microglia, pro-inflammatory signatures in were seen in early onset but not late onset AD, and in a recent analysis utilizing snRNA-seq, genotyping, and IHC, decreased numbers of phagocytic amyloid-responsive microglia were seen in APOE $\upvarepsilon$4-positive individuals with AD relative to those without this risk allele [53, 55]. In our cohort, composed of relatively young PWH not selected for the presence of AD, APOE $\upvarepsilon$4 exerted a pro-inflammatory effect in the immediate plaque microenvironment, associated with greater infiltrates of Iba1- and CD68-expressing microglia as well as GFAP-positive astrocytes (Table 5). Confirmation of this effect in a sample with predominantly diffuse plaques and large-scale perturbations of neuroimmunity due to underlying HIV disease further strengthens evidence of a mechanistic pathway whereby apolipoproteins influence microglial reactivity in the promotion or enhancement of early A$\upbeta$ plaques. It is striking that in this smaller sample selected for extant amyloid plaque, even in the face of large scale neuroimmune alterations accompanying HIV, an AD-associated risk maintained relevance to the microglial relationship with A$\upbeta$.

The other factor related to gliosis in the plaque microenvironment was sex, with men having greater gliosis than women. This counter-intuitive association of male sex with increased peri-plaque gliosis is not without precedent, particularly when one considers that this was not a cohort of individuals with AD, but a cohort of individuals with HIV and co-incident A$\upbeta$ deposition. Microglia have steroid receptors, and sex may influence microglia in variable manners throughout development; in young adults, men may have greater cortical density of activated microglia than women [56]. In one study examining brains from cognitively characterized brain donors, women had greater counts of activated microglia in the setting of AD, but conversely, in the age-matched cognitively normal group, over half of whom displayed AD neuropathology, men had greater numbers of activated microglia than women [31]. It is possible that the microenvironment relationship of male sex to greater peri-plaque inflammation in our cohort is reflective of an inherent male hyper-reactivity in early stages of abnormal A$\upbeta$ deposition, before disease progression to AD.

Finally, in the cognitively characterized sub-group of our autopsy cohort, greater levels of impairment, in domains typically associated with HIV, were associated with greater microgliosis. Furthermore, the effect was only evident in uncontrolled HIV disease. This is consonant with a literature originating before cART availability and persisting into the current therapeutic era: in individuals with uncontrolled systemic HIV, cognitive impairment is directly related to viral loads and increased brain myeloid cell infiltrates [17]. However, in individuals achieving viral suppression through cART administration, the relationship between cognition and viral loads is abrogated, as impairments persist in the absence of detectable HIV; herein we show that the lack of relationship between quantitative measures of brain microgliosis and cognitive impairment parallels this clinical dissociation. The basis of impairments in treated disease remains elusive; furthermore, the pattern of deficits correlated with large-scale frontal microgliosis may implicate other brain regions not examined in our study, as for example, medial temporal lobe structures critical to learning (memory encoding).

Limitations of our study include: the cross-sectional nature of autopsy analysis, precluding definitive cause-and effect imputation; the well-known representational bias that autopsy populations display when compared with living cohort studies; and the inability to completely balance AD risk factors (age, APOE, and diseases with inflammatory impact, such as DM) across the 3 patient groups. In examining only one frontal brain region, cognitive correlates with other neuroanatomic locations and other pathologies may have been overlooked. Finally, we did not examine the potential role of TREM2 in defining microglial phenotypes, as frequencies of AD-relevant pathogenic mutations are small; these rare mutations have demonstrated relevance to plaque-associated microglial cell phenotypes, and the soluble cleavage product of this myeloid receptor is elevated in the CSF of individuals with untreated HIV disease [53, 57]. Despite these limitations, through use of multivariable analysis, the ability to confirm a role for more common traditional AD-risk factors in the microenvironment of A$\upbeta$ plaques in a middle-aged population, while simultaneously demonstrating larger scale perturbations of neuro-immunity due to underlying viral disease, is an important step in validating a role for microglia in the earliest stages of AD neuropathology. This role may not lie in initiation of protein deposition, but rather in the promotion or enhancement of protein accumulation once initiated, in pathways mediated by known risk factors for AD, such as APOE. Future studies might therefore focus on elaborating more complete microglial phenotypes in these brain donors on a single cell basis, to understand the complex interactions of genetics and microenvironment in the early stages of neurodegenerative change.

## Supplementary Information

**Additional file 1**: Supplemental figure 1 and supplemental tables 1, 2 and 3.
