# Supplementary material for: Frontal lobe microglia, neurodegenerative protein accumulation, and cognitive function in people with HIV
Source: Acta Neuropathol Commun. 2022 May 7;10:69. doi: 10.1186/s40478-022-01375-y (PMC9080134; doi:10.1186/s40478-022-01375-y)

Supplemental table 1. Microglial cell percentage areas in sub-regions of frontal cortex defined by the presence or absence of Aβ in HIV-negative and HIV-positive individuals with and without control of plasma viremia

|  | Total sample | HIV-neg | HIV-U | HIV-D | p |
| --- | --- | --- | --- | --- | --- |
|  | n=232 | n=57 | n=81 | n=94 |  |
| CD68 cortex without Aβ^a^ | 0.0900 [0.0492,0.1607] | 0.0664 [0.0330,0.1371] | 0.0755 [0.0460,0.1350] | 0.1061 [0.0588,0.2218] | 0.0079 |
| CD163 cortex without Aβ | 0.0374 [0.0199,0.0741] | 0.0372 [0.0213,0.0697] | 0.0345 [0.0155,0.0679] | 0.0456 [0.0214,0.0802] | 0.4458 |
| Iba1 cortex without Aβ | 1.2397 [0.6770,2.0018] | 1.0468 [0.6199,1.7343] | 1.1652 [0.6129,1.9333] | 1.3756 [0.7924,2.2111] | 0.1003 |
|  | n=78 | n=25 | n=34 | n=19 |  |
| CD68 cortex with Aβ^a^ | 0.0873 [0.0420,0.1569] | 0.0828 [0.0388,0.1339] | 0.0767 [0.0311,0.1426] | 0.1393 [0.1010,0.1937] | 0.0708 |
| CD163 cortex with Aβ | 0.0590 [0.0324,0.0867] | 0.0586 [0.0376,0.0746] | 0.0401 [0.0233,0.0885] | 0.0650 [0.0506,0.1255] | 0.1565 |
| Iba1 cortex with Aβ | 1.1210 [0.6831,1.9846] | 1.4013 [0.8699,1.9381] | 1.0752 [0.4939,1.7066] | 1.1748 [0.5288,3.1133] | 0.5111 |
|  | n=56 | n=19 | n=24 | n=13 |  |
| CD68 cortex with Aβ minus without | 1.0174 (0.0078) | 1.0041 (0.0133) | 1.0182 (0.0118) | 1.0353 (0.0161) | 0.3326 |
| CD163 cortex with Aβ minus without | 1.0218 (0.0102) | 1.0159 (0.0177) | 1.0166 (0.0158) | 1.0399 (0.0214) | 0.6297 |
| Iba1 cortex with Aβ minus without | 1.0954 (0.0688) | 1.0601 (0.1198) | 1.0871 (0.1066) | 1.1613 (0.1449) | 0.8630 |

HIV-neg: HIV negative; HIV-U: HIV undetectable; HIV-D: HIV detectable; Aβ: Amyloid beta

P values refer to comparisons between HIV-neg, HIV-U, and HIV-D groups by Kruskal-Wallis tests

Median and inter quartile range displayed for all markers with exception of within-subject difference in regions with and without amyloid (last 3 rows; values translated plus one, and displayed are mean and standard error of the mean). a: HIV-D > HIV-U, HIV-neg

Supplemental table 2: Difference in mean value of area occupied by glia in plaque cores less plaque rims for the entire population examined by immunofluorescence (n=31).

|  | Cored plaque  core-rim* | p | Diffuse plaque  core-rim* | p |
| --- | --- | --- | --- | --- |
| CD68 | 5.853 (1.4624) | 0.0006 | 0.0817 (0.0333) | 0.0201 |
| Iba1 | 0.3815 (0.0522) | <0.0001 | 0.1623 (0.0271) | <0.0001 |
| GFAP | 0.2035 (0.0550) | 0.0012 | 0.0808 (0.0232) | 0.0015 |

Mean and SEM displayed (please note values in supplemental table 1 for CD68 and GFAP are medians); paired t tests utilized to test significant differences. All values log-transformed prior to analysis.

Supplemental table 3. Global and domain T scores from last cognitive assessment of PWH prospectively evaluated in MHBB study

|  |  | Adjusted r2 and p values | | | | | | | | |
| --- | --- | --- | --- | --- | --- | --- | --- | --- | --- | --- |
|  |  | All participants (n=135) | | | HIV-D (n=79) | | | HIV-U (n=56) | | |
|  | Mean (SEM) | Cortical CD68 | Cortical CD163 | Cortical Iba1 | Cortical CD68 | Cortical CD163 | Cortical Iba1 | Cortical CD68 | Cortical CD163 | Cortical Iba1 |
| Global T score | 36.2 (0.9) | **0.0887 p=0.0002** | **0.0637 p=0.0017** | **0.0300 p=0.0240** | **0.1481 p=0.0003** | **0.1183 p=0.0011** | **0.0846 p=0.0054** | -0.0107 p=0.5218 | -0.0185 p=0.9524 | -0.0184 p=0.9365 |
| Motor domain | 35.1 (1.2) | **0.0569 p=0.0085** | **0.0471 p=0.0153** | **0.0279 p=0.0494** | **0.0853 p=0.0105** | **0.0650 p=0.0228** | **0.0756 p=0.0152** | -0.0219 p=0.6515 | -0.0140 p=0.4892 | -0.0250 p=0.7559 |
| Speed of information processing | 40.3 (1.2) | -0.0063 p=0.6086 | -0.0080 p=0.7895 | -0.0080 p=0.7857 | -0.0035 p=0.3877 | -0.0136 p=0.8102 | -0.0136 p=0.8066 | -0.0222 p=0.8863 | -0.0224 p=0.9103 | -0.0127 p=0.5131 |
| Working memory | 45.4 (0.9) | -0.0076 p=0.7178 | -0.0058 p=0.5642 | -0.0044 p=0.4841 | 0.0081 p=0.2171 | 0.0090 p=0.2076 | -0.0143 p=0.8342 | 0.0010 p=0.2326 | -0.0026 p=0.3527 | 0.0060 p=0.2646 |
| Learning | 30.0 (1.1) | **0.0462 p=0.0075** | **0.0343 p=0.0184** | 0.0094 p=0.1361 | **0.0618 p=0.0166** | **0.0550 p=0.0226** | 0.0246 p=0.0918 | -0.0047 p=0.3891 | -0.0161 p=0.6902 | -0.0190 p=0.9087 |
| Recall | 30.9 (1.3) | 0.0085 p=0.1527 | -0.0069 p=0.7015 | 0.0002 p=0.3121 | 0.0126 p=0.1686 | -0.0112 p=0.6655 | 0.01177 p=0.1760 | -0.0207 p=0.9380 | -0.0170 p=0.6715 | -0.0129 p=0.5438 |
| Verbal fluency | 45.5 (1.1) | **0.0234 p=0.0512** | 0.0019 p=0.2710 | 0.0005 p=0.3062 | **0.0508 p=0.0348** | 0.0266 p=0.0955 | -0.0122 p=0.6733 | -0.0198 p=0.8628 | 0.0025 p=0.2935 | -0.0107 p=0.4959 |
| Abstraction  executive function | 42.0 (1.0) | **0.0306 p=0.0380** | -0.0012 p=0.3525 | 0.0062 p=0.1986 | 0.0423 p=0.0563 | -0.0064 p=0.4409 | -0.0006 p=0.3305 | -0.0204 p=0.7117 | -0.0226 p=0.8271 | -0.0221 p=0.7952 |

Supplemental figure 1: Representative image to demonstrate QuPath analysis of glial cells in the microenvironment of an Aβ plaque. Top row: original images with superimposed outline of the plaque using pixel classifiers applied to the AF488 Aβ channel, and the expanded outline at a radius of 50um. Bottom row: Areas of stain selected for measurement using QuPath classifiers. Panels (a, d) stain for Aβ (antibody 6E10); (b, e) stain for GFAP; and (c, f) CD68. Scale bar 50 um.

Supplemental figure 1.


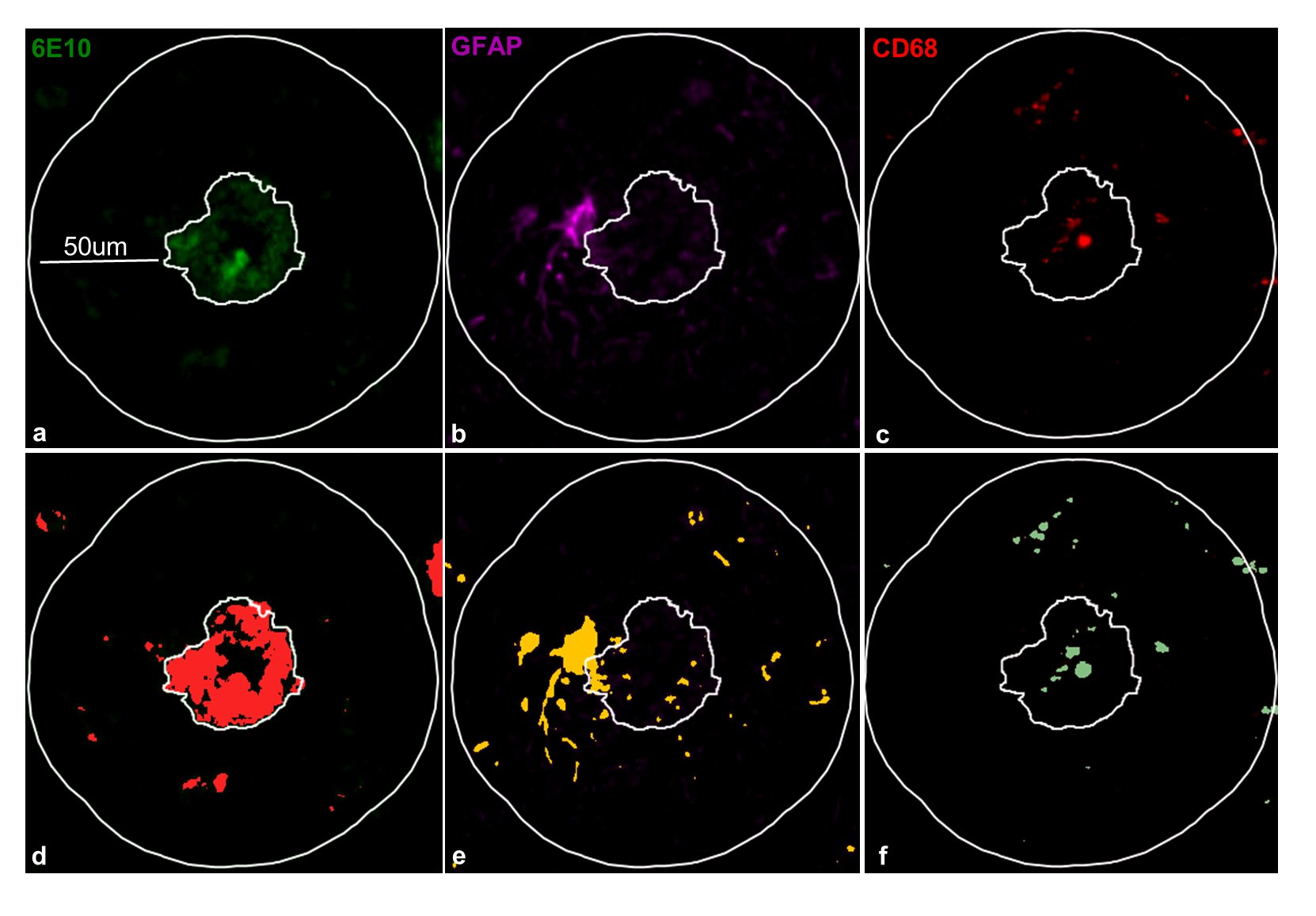

Supplement: Supplementary file 1 — Additional file 1: Supplemental figure 1 and supplemental tables 1, 2 and 3. [file 40478_2022_1375_MOESM1_ESM.docx]
